# Innovative microfossil (radiolarian) analysis using a system for automated image collection and AI-based classification of species

**DOI:** 10.1038/s41598-020-77812-6

**Published:** 2020-12-03

**Authors:** Takuya Itaki, Yosuke Taira, Naoki Kuwamori, Hitoshi Saito, Minoru Ikehara, Tatsuhiko Hoshino

**Affiliations:** 1 Geological Survey of Japan/AIST (National Institute of Advanced Industrial Science and Technology), Institute of Geology and Geoinformation, Tsukuba, Ibaraki 305-8567 Japan; 2 1st Government and Public Solutions Division, NEC Corporation, Tokyo, 108-8001 Japan; 3 Center for Advanced Marine Core Research, Kochi University, Nankoku, Kochi 783-8502 Japan; 4 Kochi Institute for Core Sample Research (KOCHI), X-star, JAMSTEC (Japan Agency for Marine-Earth Science and Technology), Nankoku, Japan

**Keywords:** Palaeoceanography, Palaeoecology

## Abstract

Microfossils are a powerful tool in earth sciences, and they have been widely used for the determination of geological age and in paleoenvironmental studies. However, the identification of fossil species requires considerable time and labor by experts with extensive knowledge and experience. In this study, we successfully automated the acquisition of microfossil data using an artificial intelligence system that employs a computer-controlled microscope and deep learning methods. The system was used to calculate changes in the relative abundance (%) of *Cycladophora davisiana*, a siliceous microfossil species (Radiolaria) that is widely used as a stratigraphic tool in studies on Pleistocene sediments in the Southern Ocean. The estimates obtained using this system were consistent with the results obtained by a human expert (< ± 3.2%). In terms of efficiency, the developed system was capable of performing the classification tasks approximately three times faster than a human expert performing the same task.

## Introduction

Microfossils that have been preserved in sediments and rocks from geological strata have been used extensively over the last 70 years for determining geological ages and in paleoenvironmental studies. The complex structure and morphology of most microfossils means that accurate identification of microfossil taxa is time consuming and requires an extensive knowledge and considerable experience to perform. However, in spite of increase needs to analyze large numbers of samples in order to obtain high-resolution records, it is concerned the scarcity of suitably trained human resources in these days.

In an attempt to address these limitations, it is expected that artificial intelligence (AI), which has progressed dramatically in recent years as computer performance has increased, could be employed in the field of microfossils classification. Deep learning using convolutional neural networks (CNN) that imitate the human brain is an application of AI that can be used to classify images after being trained on a large number of training images. Unlike traditional machine learning methods in which a person extracts the features of interest, deep learning methods based on CNNs are well suited to the classification of microfossil species with complicated structures because they automatically extract features of interest for analysis^6^. Recently, Mitra et al.^14^ demonstrated the usefulness of machine learning techniques based on CNNs to identify six species of planktic foraminifera with an accuracy greater than 80%. Hsiang et al.^5^ constructed the Endless Forams (http://endlessforams.org/) online portal, which hosts a large number of planktonic foraminiferal images that have been identified by experts, to compare the results of a CNN-based classification with the classification by humans. Furthermore, Marchant et al.^13^ reported that changes in the relative abundance of benthic foraminiferal assemblages estimated using a CNN-based classification showed good agreement with manual counts performed by humans. These recent studies have shown the effectiveness of deep learning as a method for microfossils classification.

However, when actually conducting the study, obtaining a sufficiently large number of images for compiling both training and analytical datasets can be difficult. In particular, the numerous images that are required for compiling the training dataset need to be accurately classified, which can require significant time and effort. Recently, Itaki et al.^11^ developed a system for automating the classification and accumulation of microfossil species, hereafter referred to as miCRAD (microfossil Classification and Rapid Accumulation Device) system, which is composed of three units for image collection, classification and micromanipulation. The system is based on a computer-controlled microscope/micromanipulator and a deep learning program. The automated system, which has a rapid image acquisition function combined with an accurate classification model, enables non-experts to efficiently identify large numbers of microfossils and is expected to be applied to the analysis of microfossil assemblages.

The aim of this study is to demonstrate the usefulness of the miCRAD system for revealing a microfossil assemblage using image collection and classification units of the system. Itaki et al.^11^ constructed a classification model that used microscopic images under epi-illumination to collect a single species *Cycladophora davisiana* of the siliceous microfossil radiolarians using a micromanipulator. On the other hand, we constructed classification models that employ transmitted light images in this study for implementation with the miCRAD system to automatically estimate the relative abundance (%) of *C. davisiana* (hereafter referred to as *C. davisiana*%), in an entire assemblage. We then verified a practical application of this method to estimate the *C. davisiana* % using actual down-core samples. The *C. davisiana*% has been used to classify Pleistocene sediments because it increased in subarctic regions of the ocean during glacial periods and decreased during interglacial periods (e.g.,^4,12,15^). In addition, the *C. davisiana*% has been used as a paleoceanographic indicator of intermediate water formation (e.g.,^9,16^).

## Results

This experiment was composed of following three steps: (1) collection of images of individual objects for the training dataset using the miCRAD system, (2) construction and test of the classification model based on deep learning method, and (3) estimation of the particle composition (the *C. davisiana*%) based on classification results. Details of each step are described in below.

### Image collection

**Figure 1:**
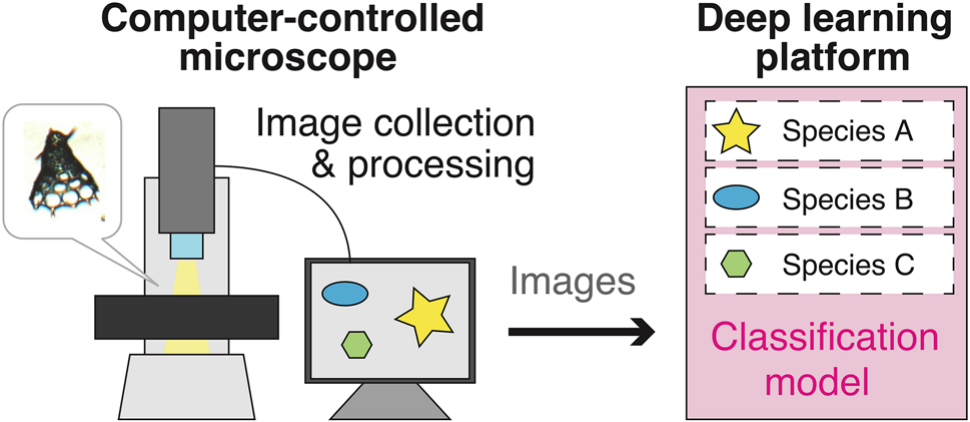
Schematic diagram of the miCRAD system used for automated image collection and classification of microfossil species.

The miCRAD system was used to collect images for compiling both the training and test datasets used in this study Fig. 1. Images were acquired using a Change Coupled Device (CCD) camera with 2 million or 5 million pixels, a × 1.5 objective and a magnification of × 4 in transmitted light mode. Binarization was used to identify individual particles in the acquired image, and the size and shape of the particles were digitized. Individual images were automatically clipped at a resolution of 280 × 280 pixels, which is sufficient for characterizing radiolarian morphology. Scanning the entire 36 × 24 mm cover glass, performing image processing, and extracting individual images of approximately 5000 objects took approximately 10 min.

### Classification models

**Figure 2:**
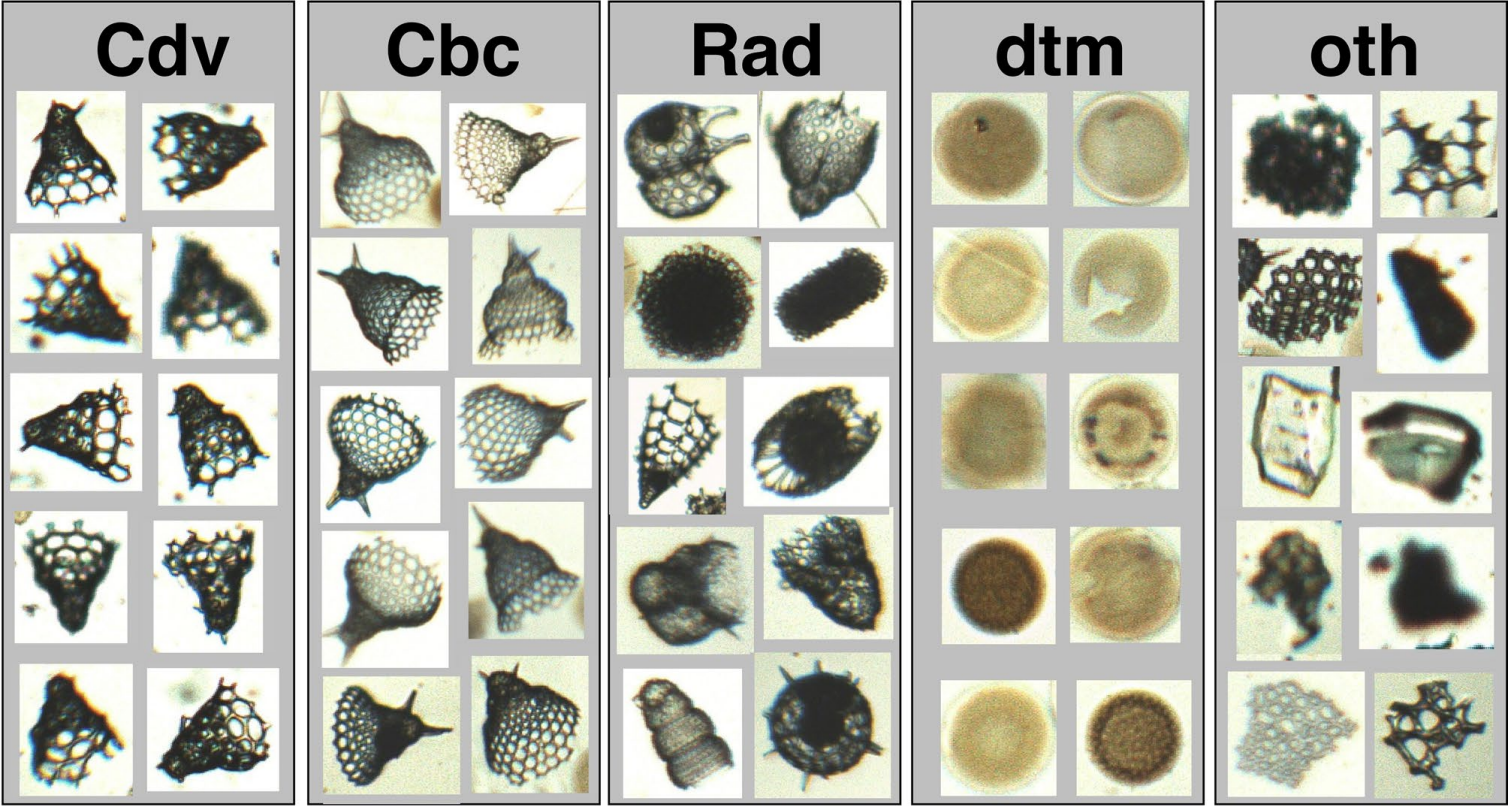
Subsets of randomly collected training data for five particle categories: *C. davisiana* [Cdv], *C. bicornis* [Cbc], all other radiolarians [Rad], diatoms [dtm], and all other particles [oth].

**Figure 3:**
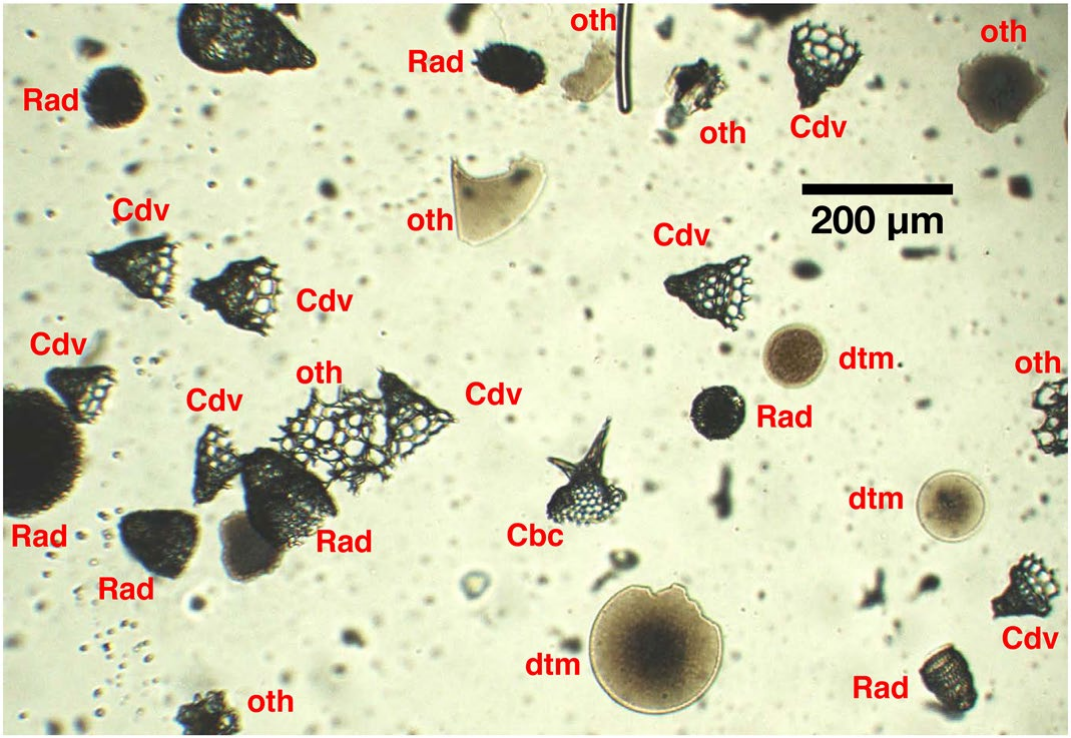
A screen shot of a scanning image showing the five particle categories in red.

The large number of individual images (> 75,000 objects) that were collected to compile the training datasets for the *C. davisiana*% classification model were sorted into five categories: “*C. davisiana*” [Cdv] containing the target species, “*Cycladophora bicornis*” [Cbc] containing taxa that are morphologically similar to *C. davisiana*, “all other radiolarians” [Rad], “centric diatoms” [dtm] and “all other particles” [oth] Figs. 2 and 3. Using these categories, two classification models were constructed using CNNs in this experiment. The models, Cdv%v2 and Cdv%v6R, were applied to images acquired at CCD camera resolutions of 2 million pixels and 5 million pixels, respectively. Table 1 shows the number of object images that were used as training dataset for each category with each model.

**Table 1:** Number of images of individual objects used as training data for each category of models Cdv%v2 and Cdv%v6R.

Category	Cdv%v2 [200 million pixels]	Cdv%v6R [500 million pixels]
*C. davisiana* [Cdv]	2992	19,007
*C. bicornis* [Cbc]	295	1,040
Other radiolarians [Rad]	4410	38,874
Diatoms [dtm]	1639	5,411
Other particles [oth]	922	2,006
Total	10,258	66,338

All the images of individual objects from the slides used for testing were acquired using the miCRAD system. These objects were classified using the two constructed models. The objects were first sorted into particle sizes ranging from 60 to 160 µm by setting an arbitrary size on GUI (Graphical User Interface) of the system, because small objects are often out of focus and, conversely, large objects often overlap with other objects. As a result, approximately 30% of the acquired object images were excluded from the analysis. Classification of about 3,000 objects took an average of 5 min.

The classification result for each object is derived from the confidence value (0 to 1.00) obtained for each object; the highest confidence value is used to assign the object to one of the five categories Table 2. For example, in the case of an image of *C. davisiana*, the results would be presented as follows: *C. davisiana* [0.87], *C. bicornis* [0.08], other radiolarians [0.03], diatoms [0.00] and other particles [0.01].

**Table 2:** An example results of the confidence values from slide code #37 obtained for each category. Bold cells indicate the highest value in the five categories. Averaged confidence values were estimated from results of all 500 objects (whole dataset is shown in Supplementary Table 1). Numbers of the value greater than confidence threshold 0.60 and 095 are also shown.

Object No	*C. davisiana* Cdv	*C. bicornis* Cbc	Other rads Rad	Diatoms dtm	Other particles oth
1	**0.87**	0.08	0.03	0.00	0.01
2	0.20	0.00	**0.79**	0.00	0.01
3	0.00	0.00	0.00	**1.00**	0.00
4	0.00	0.00	0.01	**0.98**	0.01
5	0.00	0.00	**0.92**	0.07	0.00
:					
500	0.01	**0.59**	0.03	0.09	0.28
Average	0.16	0.02	0.44	0.33	0.05
# of > 0.60	70	8	204	154	17
# of > 0.95	31	0	117	111	3

Test samples listed in Table 3 (a) and (b) were selected from samples with high and low *C. davisiana* abundance in the upper 46 cm of the core DCR-1PC used in this study to allow for extensive evaluation of *C. davisiana*% values. Slides were prepared from the same core samples as those used in the model construction, but different slides were used to obtain an independent set of images.

**Table 3:** Classification results for five test samples with model Cdv%v2 at a confidence level of 0.60 (a) and eight test samples with both models Cdv%v2 and Cdv%v6R at a confidence level of 0.95 (b). Numbers (#) indicate counts detected, and percentages (%) shows the ratio of unclassified images in the total and objects that were classified correctly in each category. Average percentage and standard deviation (S.D.) are also shown.

Model	Slide code	Core depth	Total images	Unclassified images	Category [Cdv]		Category [Cbc]		Category [Rad]		Category [dtm]		Category [oth]	
		(cm)	(#)	(%)	(#)	(correct%)	(#)	(correct%)	(#)	(correct%)	(#)	(correct%)	(#)	(correct%)
**(a)**														
Cdv%v2 Confidence threshold 0.60	1	0.6	1228	9.6	60	26.7	76	44.7	627	89.8	52	57.7	295	96.6
	13	14.5	1474	7.1	66	31.8	70	72.9	896	93.5	68	80.9	270	96.3
	21	23.6	2787	8.4	192	60.4	93	24.7	1523	79.5	311	95.5	433	92.6
	25	28.2	4005	8.0	371	77.6	78	28.2	1694	82.7	656	97.7	885	93.4
	29	32.7	4527	7.9	418	78.7	74	23.0	1566	83.8	1348	97	763	91.5
	Average %			8.2		55.1		38.7		85.9		85.8		94.1
	S.D			0.8		22.1		18.7		5.1		15.3		2.0
**(b)**														
Cdv%v2 Confidence threshold 0.95	1	0.6	1228	51.1	7	71.4	38	81.6	332	97.3	23		200	
	13	14.5	1474	45.5	11	90.9	42	69.0	536	95.7	41		173	
	21	23.6	2787	48.2	46	87.0	40	35.0	889	85.4	231		237	
	25	28.2	4005	47.9	117	93.2	34	44.1	858	90.7	515		562	
	29	32.7	4527	47.8	104	98.1	34	44.1	689	84.5	1078		459	
	33	37.3	4387	46.6	120	97.5	23	0.0	521	91.7	1529		149	
	37	41.8	4866	44.9	207	98.6	42	4.8	765	81.0	1462		167	
	41	46.3	3100	41.3	118	99.2	12	8.3	570	83.5	847		270	
	Average %			46.7		92.0		35.9		88.7				
	S.D			2.7		8.7		28.2		5.6				
Cdv%v6R Confidence threshold 0.95	1	0.6	2174	47.0	14	78.6	34	82.4	340	85.3	76		688	
	13	14.5	2387	44.5	26	88.5	52	59.6	756	90.2	80		412	
	21	23.6	5581	47.9	182	88.5	91	48.4	1428	83.0	508		697	
	25	28.2	4458	48.3	253	96.1	61	32.8	1070	82.2	396		527	
	29	32.7	6602	49.0	331	96.4	89	13.5	1596	62.4	901		452	
	33	37.3	6030	48.8	357	97.8	125	3.2	1446	72.9	798		364	
	37	41.8	6281	47.2	464	97.0	87	2.3	1466	78.9	835		466	
	41	46.3	4539	48.1	223	96.9	42	2.4	984	72.6	634		474	
	Average %			47.6		92.4		30.6		78.4				
	S.D			1.3		6.3		28.5		8.2				

Table 3 (a) shows the classification results obtained using model Cdv%v2 for five test samples with a confidence level of 0.60 taken as the threshold value. In the case of a confidence level of 0.6, approximately 90% of the objects exceeded the threshold and were classified successfully. This extraction rate is larger than approximately 50% at the confidence level of 0.95 on the same slide as shown in Table 3 (b). Radiolarians, diatoms and other particles were classified with an accuracy of at least 80%, on average, even with a relatively low confidence level, implying that it was generally possible to distinguish between images of radiolarians and other particles. However, the classification accuracy for *C. davisiana* was low, ranging from 26.7 to 78.7% (55.1% on average), implying that these model conditions were not suitable for estimating the relative abundance of this species.

Table 3 (b) shows classification results for eight test samples obtained using models Cdv%v2 and Cdv%v6R and a confidence level of 0.95. Briefly, *C. davisiana* was classified with a high level of accuracy using both models,71.4–99.2% (92.0% on average) using model Cdv%v2 and 78.6–97.8% (92.4% in average) for model Cdv%v6R. Although slide code #1 shows a relatively low accuracy for *C. davisiana* (< 80%) due to the small detected count, this is not a serious problem for calculating the *C. davisiana*% because of the small contribution of this value to the overall assemblage.

The classification accuracy for *C. bicornis* was generally low for both models, ranging from 0.0–81.6% (35.9% on average) with model Cdv%v2 and 2.3–82.4% (30.6% on average) with model Cdv%v6R. In particular, accuracy was markedly low for three samples (# 33, 37 and 41) due to a small number of detections. However, the misclassified *C. bicornis* image does not include *C. davisiana*, and conversely the misclassified *C. davisiana* image does not include *C. bicornis*, which means that species with a similar structure can still be distinguished by these models.

The accuracy for the other radiolarians was 81.3–97.3% (88.7% on average) estimated by Cdv%v2 and 62.4–90.2% (78.4% on average) by Cdv%v6R, with the latter being somewhat low. The low accuracy obtained using Cdv%v6R was largely attributed to the model misclassifying diatom fragments. In the future, to improve the accuracy of the model, it will be necessary to evaluate and update these misclassified objects.

### Practical test for the *C. davisiana*% curve

In this section, the constructed classification models are tested for practical use based on core DCR-1PC collected from the Southern Ocean.

**Figure 4:**
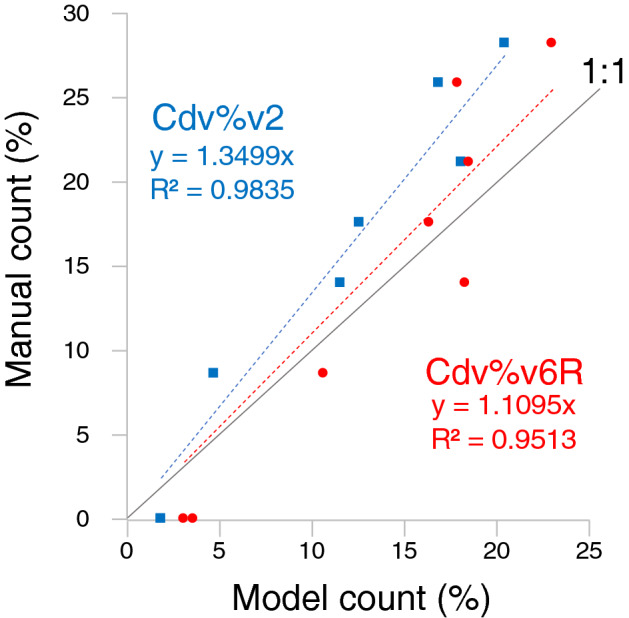
Plots of the *C. davisiana*% for the manual count vs the model count.

The *C. davisiana*% in the down-core test samples was calculated from objects that had been classified into each of the five categories using the two models (Supplementary Table 2. Analysis of 8 test samples from the upper 46 cm of the core using the models yielded the *C. davisiana*% values that ranged from 0 to 23%, which showed a high correlation with manual count data, r = 0.972 and r = 0.942 for models Cdv%v2 and Cdv%v6R, respectively Fig. 4. Such high correlations of the *C. davisiana*% between manual counting and results from both models are consistent with the trend for additional core samples from the upper 46 cm (17 samples for Cdv%v2 and 11 samples for Cdv%v6R) (Supplementary Table 2 implying that is expected to be highly reproducible. However, the slope of the regression line was slightly larger than 1 meaning that the model estimates were underestimated due to relatively lower accuracy of other radiolarians [Rads] than those of *C. davisiana* [Cdv].

**Figure 5:**
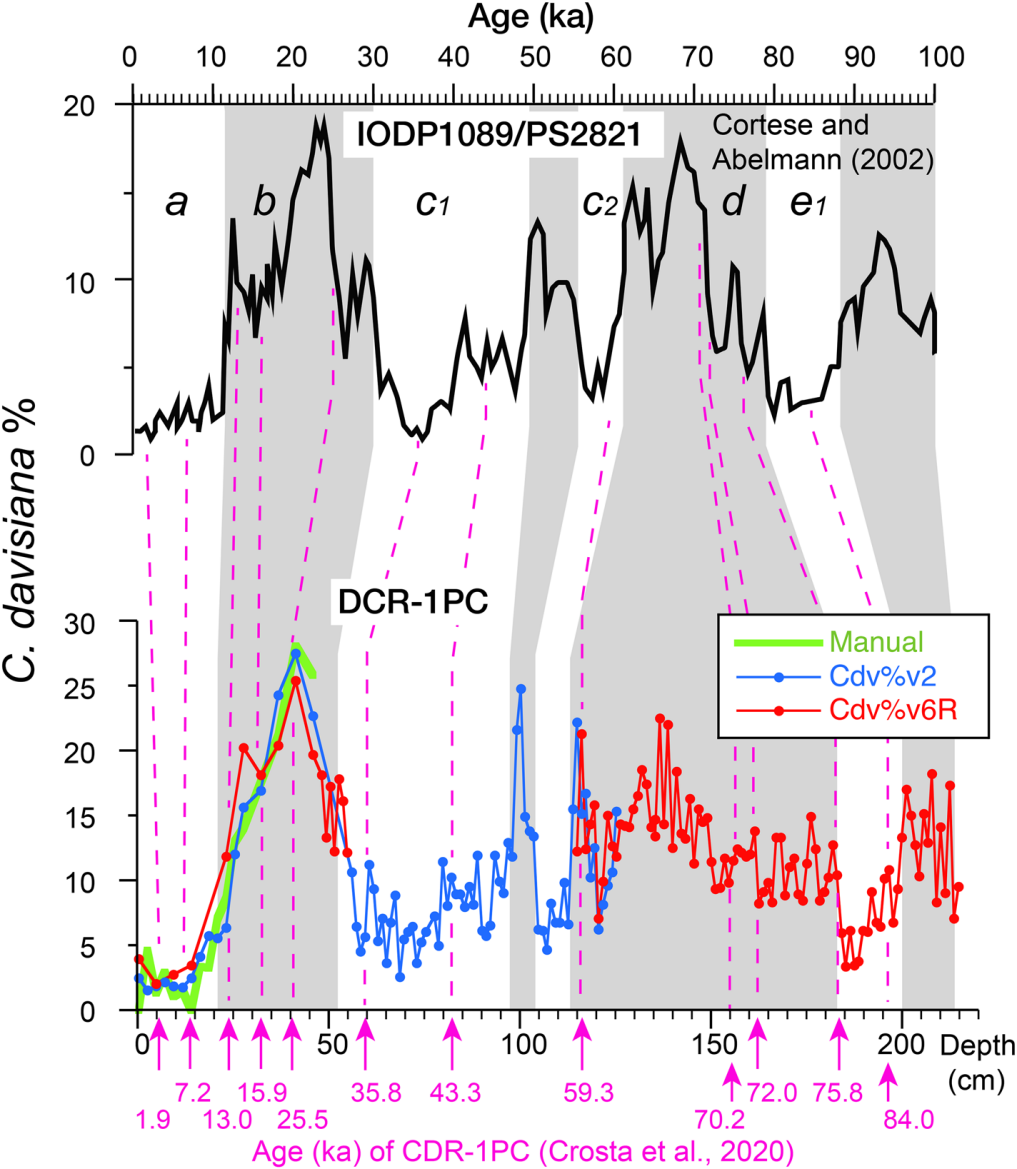
The *C. davisiana*% curves for the manual count of the upper 46 cm of the core (green) and those estimated from model data to a depth of 216 cm (blue and red). Age model of core DCR-1PC is from Crosta et al.^3^ (pink arrows in the lower graph panel). Correlation with results from sites PS2821/ODP 1089 by Cortese and Abelmann^2^ is also shown (dashed pink lines). The *C. davisiana* zones *a, b, c…e*_*1*_ of Hays et al.^4^ are indicated in the upper graph panel and correlated with gray bands.

Using the correlation equations shown in Fig. 4, the data obtained by multiple classification models having different tendencies can be calibrated as actual count data. Figure 5 shows the calibrated *C. davisiana*% curve for the upper 216 cm applied to the continuous data for the DCR-1PC down-core samples obtained by the miCRAD system. The calibrated values for both models corroborate each other, and the error associated with human count data for the upper 46 cm was within ± 3.2% (standard deviation). Furthermore, when data collection by the models was expanded to the upper 216 cm of the core, marked fluctuations ranging between 2 and 25% were observed Fig. 5.

### Discussion

The *C. davisiana*% curve, which shows high values during glacial periods and low values during interglacial periods, has been widely used in studies involving Pleistocene age determination in carbonate-poor sediments in the Southern Ocean (e.g.,^1,4^). The *C. davisiana*% curve obtained from core DCR-1PC shows low values through the upper 20 cm and high values between 20 and 60 cm, which corresponds to zones “*a*” [Holocene] and “*b*” [Last glacial period] of *C. davisiana* stratigraphy^4^. In Fig. 5, the *C. davisiana*% variations detected by the miCRAD system are compared with results obtained from Ocean Drilling Program site 1089/PS2821 in the Atlantic sector of the Southern Ocean^2^. Despite the considerable distance about 3,000 km between both sites (Supplementary Fig.1), their stratigraphic characteristics are very similar. Since this correlation is consistent with an age model applied to core DCR-1PC by Crosta et al.^3^, the *C. davisiana*% data collected by the miCRAD system is considered to be comparable with analysis by a human expert.

Furthermore, considerable increases in time efficiency can be achieved using this system. The time required for scanning, image processing and classification of microfossils using the miCRAD system depends on the density of particles mounted on the slide. In the case of the core DCR-1PC samples used in this study, approximately 1000 radiolarians can be detected in a slide area of 18 × 24 mm (about half the area of a cover glass), and the analysis can be completed within 10 min. On the other hand, since it typically takes an expert about 30 min to count the same number of radiolarians under a microscope, the time required for data acquisition using the system is three times faster than that required by a human. Furthermore, continuous data acquisition is possible as there is no human fatigue associated with microscope observations. At the Geological Survey of Japan, one technician can operate two miCRAD systems simultaneously, which means that throughput can be doubled, and a mass production system has been established.

The challenge is to build a more efficient model. The two classification models constructed in this study had a high accuracy for *C. davisiana* when the confidence threshold was set to 0.95, but approximately half of the images obtained by the miCRAD system could not be classified for confidence thresholds below this value. On the other hand, when the confidence threshold was lowered to 0.60, the number of unclassified images was reduced to approximately 10%, but accuracy also decreased. As reported by Itaki et al.^11^, more efficient data acquisition requires that a classification model be developed that can achieve a high accuracy, even with a low confidence level.

As another method for evaluating particle composition, it is expected estimation from averaged confidence values for each of the five categories could be used, as demonstrated by Shoji et al.^17^ who classified the composition of volcanic ash particles. In this method, the confidence values obtained for each category are assigned to images of each particle Table 2. For example, when the confidence value of object No. 1 is assigned as *C. davisiana* [0.87], *C. bicornis* [0.08], other radiolarians [0.03], diatoms [0.00] and other particles [0.01], the category with the highest confidence value, i.e. *C. davisiana*, would be excluded from the classification as the obtained value is below the threshold of 0.95. However, the composition of the constituent particles in the sample can be estimated from the averaged value of the confidence values for all particles in each category. Using 500 objects from slide code #37 shown in Table 2 (whole 500 data is shown in Supplementary Table 1) as an example, the relative contribution of *C. davisiana* to the total radiolarians estimated from ratios of averaged confidence values for categories of *C. davisiana* [0.16], *C. bicornis* [0.02] and other radiolarians [0.44] is 26.1%, which is consistent with a result of 28.2% obtained by a human expert. Although this method involves uncertainty in classification accuracy because no classification threshold is provided, efficient data acquisition is possible because no images are excluded in the determination. In the future, we plan to examine the potential of such a method and may update the miCRAD software accordingly.

In this study, although the models were successfully designed to estimate the *C. davisiana*%, it is still restricted to apply a model that is capable of analyzing more complex assemblages by classifying multiple species simultaneously. Furthermore, fossil and particle compositions are usually various in different regions and periods. For further efficiency, many categories of training data are needed, but accuracy tends to decrease as the number of categories increases in currently used programs. Therefore, not all work by experts at this stage can be replaced by this system. To solve this problem, it is expected that more training data will be collated using a variety of AI technologies.

We have already conducted classification tests on microfossils other than radiolarians using deep learning methods (e.g.,^6^). The high classification accuracy achieved for microfossils with complicated features using deep learning methods also suggests that these methods can be applied to a variety of other kinds of fine particles. For example, Shoji et al.^17^ classified the particle composition of volcanic ash using a CNN, but the miCRAD system could be adapted to analyze data from volcanic eruptions. In addition, the system could also be applied to the detection of contaminants in the fields of food safety and medicine.

## Methods

### Sample procedure

Sediment core DCR-1PC used in this study was recovered from the Del Caño Rise (46°01.34′S, 44°15.24′E, water depth: 2,632 m) in the Indian sector of the Southern Ocean during the KH-10-7 cruise of R/V *Hakuho-maru* (Supplementary Fig. 1). The core is composed of alternating homogeneous nannofossil oozes and diatom oozes. The slide for radiolarian observation was prepared from samples sliced at 1 cm intervals using standard methods described in Itaki et al.^10^. Briefly, slide preparation involved the following steps: (1) Remove carbonates and organic matter with hydrochloric acid and hydrogen peroxide,(2) Wet sieve with a screen with a 45 μm mesh size; (3) Concentrate siliceous fossils by the method of Itaki^8^,(4) Disperse particles as much as possible on the slide so that particles do not overlap; (5) After drying the particles on the slide, mount them with an optical adhesive.

To obtain the training data images, eight slides were prepared from the upper 46 cm of the core, and slides for practical testing were made from continuous samples down to 216 cm.

### Image collection and classification

Image collection and classification in this study were carried out using the miCRAD system designed by Itaki et al.^11^, which is a system that connects a deep learning software “RAPID Machine Learning” (NEC Corp., Tokyo, Japan) to a microscope with a computer-controlled, motorized XY stage and a micromanipulator “Collection Pro” (Micro Support Co., Ltd. , Shizuoka, Japan) referred to as an automatic zircon separator in Isozaki (2018). The program is customized to control the hardware and facilitate effective image collection. The image collection and classification units of the miCRAD system were used in this study.

Although clearer images could be acquired using the automatic focus composition function of the miCRAD system, the time required to acquire clearer images is approximately three times that of normal mode. In order to save time for image collection, we decided to take images without this focus composition function, and to add unfocused images to the training dataset to facilitate the classification of even unclear images.

For the training dataset, a large number of individual images of objects collected using the miCRAD system was divided into five categories: *C. davisiana* (Cdv), *C. bicornis* (Cbc), all other radiolarians (Rad), diatoms (dtm), and all other particles (oth). For categories with a small number of individual images, data was amplified by rotating the images. Based on these training data, a CNN classification model was automatically constructed using a deep learning software platform “RAPID machine learning” (NEC Corp.), which can be easily operated on the GUI. Generally, the initial generated model has a low accuracy, so we tried to improve accuracy in a stepwise manner by trial and error, such as by changing categories and amplifying training data.

Image acquisition and classification process by miCRAD system are all operated on the GUI of the system. Here is a brief description of the procedure. (1) Set the slide on the automated XY stage and adjust the focus (if necessary, focus composition). (2) Binarization threshold adjustment by contrast, light intensity, color tone, brightness, size, etc., to better extract object images from the scanned image (settings can be saved for observation under the same conditions). (3) Specify the observation area of the slide and scan it. (4) Selecting a classification model built by "RAPID Machine Learning"  to automatically classify the clipped image of the objects. (5) Classification results are saved as images and a CSV file.

Based on the classification results, the relative abundance of *C. davisiana* against total radiolarians was calculated as following equation:

$$C. \, davisiana\,\% = Cdv/\left( {Cdv + Cbc + Rad} \right) \times {1}00$$

where *Cdv*, *Cbc* and *Rad* are count values estimated by the miCRAD system for *C. davisiana*, *C. bicornis* and all other radiolarians, respectively. As a standard, we used the *C. davisiana*% obtained by manual counting of radiolarians in the same sample by an expert; counted number of the standard data was more than 300 individuals (305 to 551 individuals).

## Supplementary information

Supplementary Legends.

Supplementary Table.

Supplementary Table.

Supplementary Figure.
