# Supplementary material for: Innovative microfossil (radiolarian) analysis using a system for automated image collection and AI-based classification of species
Source: Sci Rep. 2020 Dec 3;10:21136. doi: 10.1038/s41598-020-77812-6 (PMC7713231; doi:10.1038/s41598-020-77812-6)
Supplement: Supplementary file 1 — Supplementary Legends. [file 41598_2020_77812_MOESM1_ESM.docx]

Supplementary Table 1. Whole dataset of Table 2. Confidence values of 500 objects from sample Edu#37 obtained for each category.

Supplementary Table 2. The *C davisiana*% estimated by manual counts of the upper 46 cm of the core and that estimated from model data to a depth of 216 cm.

Supplementary Figure 1. Locations of core site DCR-1PC (this study) and ODP site 1089/PS2821 (Cortese and Abelmann, 2002).
