# Supplementary figures and images for: Innovative microfossil (radiolarian) analysis using a system for automated image collection and AI-based classification of species
Source: Sci Rep. 2020 Dec 3;10:21136. doi: 10.1038/s41598-020-77812-6 (PMC7713231; doi:10.1038/s41598-020-77812-6)

Supplementary Figure 1

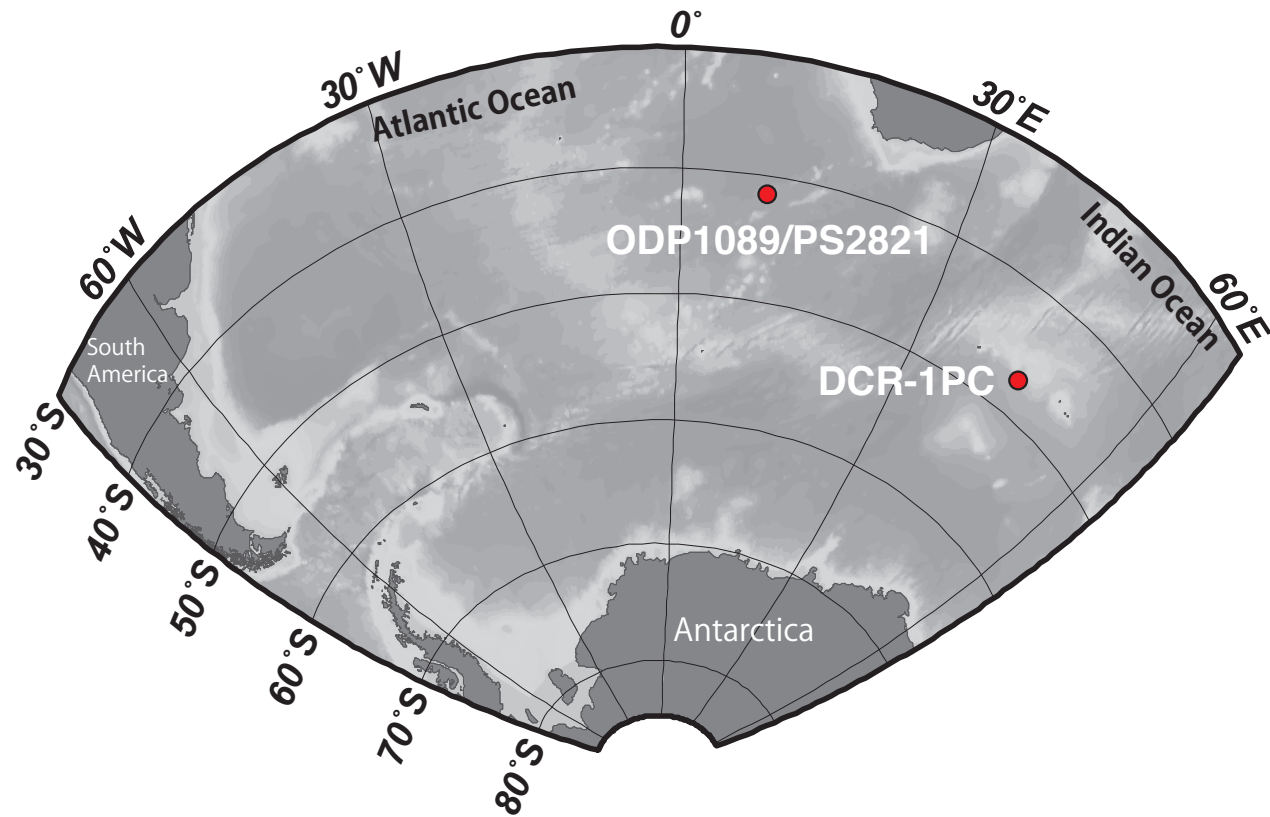

Supplement: Supplementary file 4 — Supplementary Figure. [file 41598_2020_77812_MOESM4_ESM.pdf]
